# Determining the per capita consumption of Tah-dig in the Iranian food table

**DOI:** 10.1186/s12889-025-25551-6

**Published:** 2025-12-08

**Authors:** Yeganeh Mazaheri, Parisa Sadighara, Samira Shokri, Fatemeh Salmani, Kamiar Mahmoudifar, Fatemeh Hoseinzadeh-Chahkandak, Ensiyeh Norozi, Tayebeh Zeinali

**Affiliations:** 1 Department of Environmental Health Engineering, Food Safety Division, School of Public Health, Tehran University of Medical Sciences, Tehran, Iran; 2 Nutritional Health Research Center, Lorestan University of Medical Sciences, Khorramabad, Iran; 3 Department of Epidemiology and Biostatistics, School of Health, Geriatric Health Research Center, Birjand University of Medical Sciences, Birjand, Iran; 4 Department of Nutrition, School of Medicine, Mashhad University of Medical Sciences, Mashhad, Iran; 5 Department of Nutrition and Food Hygiene, School of Health, Geriatric Health Research Center, Birjand University of Medical Sciences, Birjand, Iran; 6 Department of Public Health, School of Health, Social Determinants of Health Research Center, Birjand University of Medical Sciences, Birjand, Iran

**Keywords:** Per capitaTah-dig consumption, Edible oils, Bottom pot rice, Bread, Potato

## Abstract

**Background:**

The traditional Iranian dish, Tah-dig, holds significant cultural and culinary importance. Tah-dig forms as a crispy layer of rice, bread, or potatoes at the bottom of the cooking pot, offering a delightful textural contrast. This study aims to determine the per capita consumption of Tah-dig in Iran.

**Methods:**

Data on the per capita consumption of various types of Tah-dig were collected from 1,664 participants using a questionnaire administered between 2021 and 2023.

**Results:**

The findings indicate that the most frequently consumed Tah-dig type in Iran is rice-based, with a mean consumption of 0.56 serving/day. Bread-based Tah-dig follows with a mean consumption of 0.52 serving/day, which is higher than the means for potato and macaroni-based Tah-dig, respectively.

**Conclusions:**

The overall consumption of Tah-dig among Iranians is notably high. Rice-based Tah-dig demonstrates the highest per capita consumption. Given the potential formation of harmful toxins in Tah-dig during cooking, further investigation into its safety is warranted.

**Supplementary Information:**

The online version contains supplementary material available at 10.1186/s12889-025-25551-6.

## Background

Ensuring food security and proper nutrition is critical for the health and well-being of any population. Developing comprehensive strategies regarding food quality, per capita consumption, public health, dietary guidelines, food composition, and safety measures is vital for national health. Policymakers utilize information about food consumption and per capita consumption for analyzing the socio-economic conditions, food system legislation and health policy development [1–3].

Food consumption patterns reveal socio-economic disparities, generational transitions, and health practices. Studies show that higher socio-economic status correlates with healthier dietary habits, whereas lower socio-economic groups consume more traditional or processed foods. This generational shift toward modern, processed diets among younger demographics raises concerns regarding long-term health impacts [4–7]. Iran, located in the Middle East, is presently experiencing a rapid nutritional transition marked by a shift from traditional, health-promoting dietary practices to more modern patterns that are often less beneficial to health. This transition is accompanied by a significant rise in the prevalence and burden of non-communicable diseases, particularly among the adult population [8] Dietary habits in Iran also exhibit significant variations shaped by socioeconomic factors, generational changes, and health-related practices [9].

Among traditional Iranian dishes, *Tah-dig*—derived from the Persian words *tah* (bottom) and *dig* (pot)—is iconic and widely consumed both domestically and across the Middle East [10]. It is a carbohydrate-rich, fried food [11], prepared by cooking rice, bread, or potatoes to form a golden crust at the bottom of the pot [10]. Popular variations include bread-based and potato-based *Tah-digs*, as well as other types made with ingredients like saffron, lettuce, yogurt [10].

The preparation of Tah-dig requires significant use of oil, making it a fat-rich dish. The high temperatures during cooking, coupled with the organic materials in *Tah-dig*, create conditions conducive to the formation of Polycyclic Aromatic Hydrocarbons (PAHs) [12], which are toxic, carcinogenic compounds linked to DNA damage and cancer [13, 14]. Considering the Akbari-Adergani et al. study, the maximum PAH contamination was detected in bread and potato Tah-digs [12]. Additionally, the heat-induced oxidation of oils produces acrylamide, a carcinogenic contaminant [15, 16].

Despite the popularity of Tah-dig, comprehensive data on its per capita consumption remains unavailable. Given the public health implications of PAH and acrylamide contamination, this study seeks to estimate the per capita consumption of Tah-dig in Iran to better understand the exposure levels and address potential health concerns. The findings are anticipated to enhance our understanding of exposure risks and inform food safety policies aimed at safeguarding consumer health.

## Methods

### Study plan and participants

This descriptive cross-sectional study was conducted between 2021 and 2023. A minimum number of 1645 participants were calculated as adequate sample size. Sample size was determined based on the study of Karimi et al. (2022) [11]. Studies show that 40% of households consume rice seven times a week, and 82.5% of households always had Tah-dig with their rice. Additionally, households consumed various types of Tah-dig, including bread, rice, and potato, at rates of 41.4%, 37.2%, and 21.4% respectively. Furthermore, 98.2% of the participants expressed a preference for eating fried Tah-dig. The largest sample size was obtained based on 41.4% based on following formula:

$$\:n=\frac{{\left({z}_{1-\frac{\alpha\:}{2}}+{z}_{1-\beta\:}\right)}^{2}p\left(1-p\right)}{{d}^{2}}=\frac{{\left(1.96+0.84\right)}^{2}0.414\left(1-0.414\right)}{0.03{4}^{2}}\approx\:1645$$

In which: n is for sample size; $\:{z}_{1-\frac{\alpha\:}{2}}$ stand for confidence level; $\:{z}_{1-\beta\:}$ stand for power; p is for prevalence and d is for precision.

Questionnaire was viewed by more than 2000 people. Sampling was carried out using convenience sampling. A prepared checklist to measure Tah-dig consumption was distributed online through social media platforms such as Telegram, WhatsApp, Instagram, emails, and other digital channels across various regions of Iran. Participants were asked to share the questionnaire with two additional family members. Completing the questionnaire required approximately 15 min.

#### Inclusion criteria

The participants consisted of Iranian individuals who had internet access, consumed Tah-dig, were proficient in Persian, and were willing to contribute to the research.

#### Exclusion criteria

The following people were not included in the study: [1] The people who did not consume Tah-dig and could not understand Persian language [2]. Those that couldn’t use smartphones or did not have access to the internet. Moreover, the questionnaires that did not completely filled out especially the section of serving size of consuming Tah-digs were excluded from the study.

### Instrument

To estimate the per capita consumption of *Tah-dig*, a comprehensive questionnaire was developed. The questionnaire was divided into three sections: The first part consists of demographic questions about age, marital status, and income evaluation. The second part contained questions about the cooking methods of Tah-dig such as the type of cooking oil and duration of cooking. The third part was consumption patterns: Examined the frequency and serving sizes of different Tah-dig types, including bread-, potato-, rice-, and macaroni-based variants. One serving size is equal to a square of 10 centimeter × 10 centimeter. The actual consumption of Tah-dig was estimated by multiplying the number of servings size by the frequency of consumption per day. For instance, an individual who consumed one serving size of Tah-dig once per week was assigned a daily intake value of 0.14 servings. Similarly, an individual who consumed two servings once per week had a calculated daily intake of 0.28 serving (i.e., 2 × 0.14). The average daily consumption across all participants was subsequently calculated and reported as per capita intake. The supplementary file includes the used questionnaire.

The face validity of the initial items was evaluated by ten Tah-dig consumers to assess the simplicity and difficulty of the items. Additionally, the necessity of each item was analyzed by ten experts using the Lawsche table to determine the Content Validity Ratio (CVR) [17]. Items with CVR > 0.62 were deemed necessary. The Content Validity Index (CVI) was calculated based on Lynn’s pattern [18], using a four-point scale to assess simplicity and relevance. Items with a CVI exceeding 0.78 were accepted [18]. All items achieved a CVR above 0.62 and a CVI above 0.80 were included in the analysis.

### Ethical consideration

This study adhered to the ethical principles outlined in the Declaration of Helsinki regarding research involving human participants. Ethical approval was obtained from the Ethics Committee of Birjand University of Medical Sciences (IR.BUMS.REC.1400.380). Written informed consent was secured.

### Statistical analysis

All statistical analysis was performed using SPSS version 22 (SPSS, Inc., Chicago, IL). Qualitative variables were presented as frequencies and quantitative variables were reported as means and standard deviations (SD). Per capita consumption of Tah-dig was compared by type of oil used with Student’s t-test to compare users and non-users of Tah-dig. One-way analysis of variance (ANOVA) along with Tukey’s post-hoc test was used to analyze per capita consumption by time range for cooking. A p-value < 0.05 was considered statistically significant.

## Results

A total of 1,701 structured questionnaires were completed across various regions of Iran. Of these, 1,664 respondents provided data for the serving size section. The sample was predominantly female (69.7%), with a mean age of 32.49 years. Summary demographic characteristics of the participants are presented in Table 1.

**Table 1:** Demographic characteristics of the participants of the study

		Number	Percent
Gender	Male	505	30.3
	Female	1159	69.7
Income	Low	483	29.3
	Medium	868	52.7
	High	296	18.0
Age categories	Children and Youth < 24	435	26.8
	Young Adults 25–44	854	52.6
	Middle Age 45–59	292	18.0
	Elderly >60	44	2.7
		Mean ± SD	Median (IQR)
Age		32.49 ± 11.53	32 (17)

*SD* Shows standard deviation, *IQR* Interquartile range

Table 2 reports the average per capita daily consumption of different types of Tah-dig. The mean daily intake per capita was approximately 0.56 serving/day for rice Tah-dig, 0.52 serving for bread Tah-dig, 0.28 serving for potato Tah-dig, and 0.12 serving for macaroni Tah-dig. Age-based analysis [19, 20], (Table 3) revealed significant differences in the consumption of bread and potato Tah-dig across age groups. Older adults and middle-aged individuals consumed less bread Tah-dig compared to children and Youth. Similarly, elderly participants exhibited lower consumption of potato Tah-dig than all other age groups, including youth, young adults, and middle-aged individuals. Gender differences were also observed (Table 4), with female respondents reporting higher consumption of macaroni Tah-dig than their male counterparts.

**Table 2:** Per capita consumption of different Tah-digs (serving/day)

	Mean	SE	Median	IQR	95% Confidence Interval for Mean	
					Lower Bound	Upper Bound
Bread	0.52	0.10	0.06	0.49	0.32	0.73
Potato	0.28	0.06	0.08	0.15	0.16	0.40
Rice	0.56	0.10	0.03	0.50	0.36	0.76
Macaroni	0.12	0.04	0.01	0.08	0.045	0.19

*SE* Standard error of mean, *IQR* Interquartile range

**Table 3:** Comparison of per capita consumption of different Tah-digs (serving/day) by age categories

Per capita consumption	Age	Mean	SE	Median	IQR	*P*-value	Post-Hoc
Bread	Children and Youth < 24	0.96	0.27	0.08	0.5	0.024	Elderly - Children and Youth: *P* = 0.033 Middle Age- Children and Youth: *P* = 0.012
	Young Adults 25–44	0.31	0.07	0.06	0.49		
	Middle Age45-59	0.39	0.25	0.03	0.23		
	Elderly >60	0.01	0.006	0.007	0.02		
Potato	Children and Youth < 24	0.26	0.11	0.06	0.15	0.017	Elderly - Middle Age: *P* = 0.031 Elderly - Children and Youth: *p* = 0.005 Elderly - Young Adults: *p* = 0.004
	Young Adults 25–44	0.28	0.06	0.08	0.27		
	Middle Age45-59	0.37	0.26	0.08	0.13		
	Elderly >60	0.01	0.006	0.007	0.02		
Rice	Children and Youth < 24	0.62	0.21	0.03	0.25	0.365	-
	Young Adults 25–44	0.55	0.12	0.07	0.50		
	Middle Age45-59	0.54	0.28	0.08	0.49		
	Elderly >60	0.13	0.12	0.007	0.38		
Macaroni	Children and Youth < 24	0.10	0.02	0.01	0.1	0.06	-
	Young Adults 25–44	0.08	0.01	0.03	0.08		
	Middle Age45-59	0.33	0.25	0.01	0.08		
	Elderly >60	0.004	0.002	0.003	0.01		

*SE* Standard error of mean, *IQR* Interquartile range

**Table 4:** Comparison of per capita consumption of different Tah-digs (serving/day) by gender

Per capita consumption	Gender	Mean	SE	Median	IQR	*P*-value
Bread	Male	0.50	0.16	0.08	0.49	0.23
	Female	0.53	0.14	0.03	0.34	
Potato	Male	0.28	0.06	0.11	0.27	0.85
	Female	0.28	0.08	0.03	0.15	
Rice	Male	0.41	0.13	0.06	0.5	0.25
	Female	0.65	0.14	0.03	0.5	
Macaroni	Male	0.11	0.02	0.03	0.14	0.032
	Female	0.12	0.05	0.01	0.08	

*SE* Standard error of mean, *IQR* Interquartile range

Table 5 compares per capita consumption of Tah-dig types based on cooking duration. A statistically significant difference was found only in the consumption of bread Tah-dig between the 15-minute cooking time and the longer durations of 30 and 45 min (*p* < 0.05).

**Table 5:** Comparison of per capita consumption based on cooking time

Per capita consumption	Time	Mean	SE	Median	IQR	*P*-value	Post-Hoc
Bread	45 min	0.66	0.18	0.08	0.50	0.024	15–30 min: *P* = 0.023 15–45 min : *P* = 0.007
	30 min	0.40	0.17	0.03	0.15		
	15 min	0.51	0.41	0.01	0.11		
Potato	45 min	0.26	0.08	0.08	0.16	0.274	-
	30 min	0.35	0.15	0.04	0.23		
	15 min	0.07	0.03	0.02	0.11		
Rice	45 min	0.64	0.16	0.07	0.50	0.434	-
	30 min	0.51	0.19	0.03	0.50		
	15 min	0.24	0.18	0.02	0.11		
Macaroni	45 min	0.15	0.08	0.01	0.08	0.374	-
	30 min	0.09	0.02	0.01	0.08		
	15 min	0.04	0.01	0.01	0.08		

Number of Consumer: 45 min: 826; 30 min: 538; 15 min: 102

*SE* Standard error of mean, *IQR* Interquartile range

Table 6 presents consumption patterns according to the type of oil used in preparation. Overall, individuals who did not use oil reported higher per capita consumption of Tah-dig than those who did. Notably, significant differences in rice Tah-dig consumption were observed between oil users and non-users for two specific types of vegetable oils (*p* < 0.05).

**Table 6:** Comparison of per capita consumption based on type of used oil

Oil	Tah-dig	Use of oil	Mean	SE	Median	IQR	*P*-value
Olive, Sesame and Canola oil	Bread	Non-user	0.64	0.14	0.06	0.49	0.550
		User	0.15	0.04	0.03	0.15	
	Potato	Non-user	0.33	0.08	0.08	0.15	0.854
		User	0.14	0.03	0.06	0.15	
	Rice	Non-user	0.60	0.12	0.03	0.50	0.031
		User	0.45	0.17	0.03	0.50	
	Macaroni	Non-user	0.14	0.05	0.02	0.08	0.194
		User	0.05	0.01	0.00	0.08	
Butter and Ghee oil	Bread	Non-user	0.59	0.12	0.08	0.49	0.972
		User	0.13	0.05	0.01	0.14	
	Potato	Non-user	0.28	0.07	0.07	0.15	0.592
		User	0.26	0.07	0.11	0.27	
	Rice	Non-user	0.62	0.12	0.03	0.50	0.106
		User	0.27	0.12	0.01	0.15	
	Macaroni	Non-user	0.13	0.04	0.01	0.08	0.658
		User	0.06	0.01	0.01	0.10	
Corn, sunflower oil	Bread	Non-user	0.58	0.14	0.04	0.44	0.136
		User	0.35	0.13	0.05	0.49	
	Potato	Non-user	0.24	0.06	0.06	0.15	0.080
		User	0.38	0.15	0.01	0.24	
	Rice	Non-user	0.56	0.13	0.03	0.50	0.030
		User	0.57	0.16	0.08	0.50	
	Macaroni	Non-user	0.13	0.05	0.01	0.08	0.154
		User	0.09	0.02	0.02	0.14	

Non-user Number: 1355 (80.7%); User: 325 (19.3%)

*SE* Standard error of mean, *IQR* Interquartile range

**Fig. 1:**
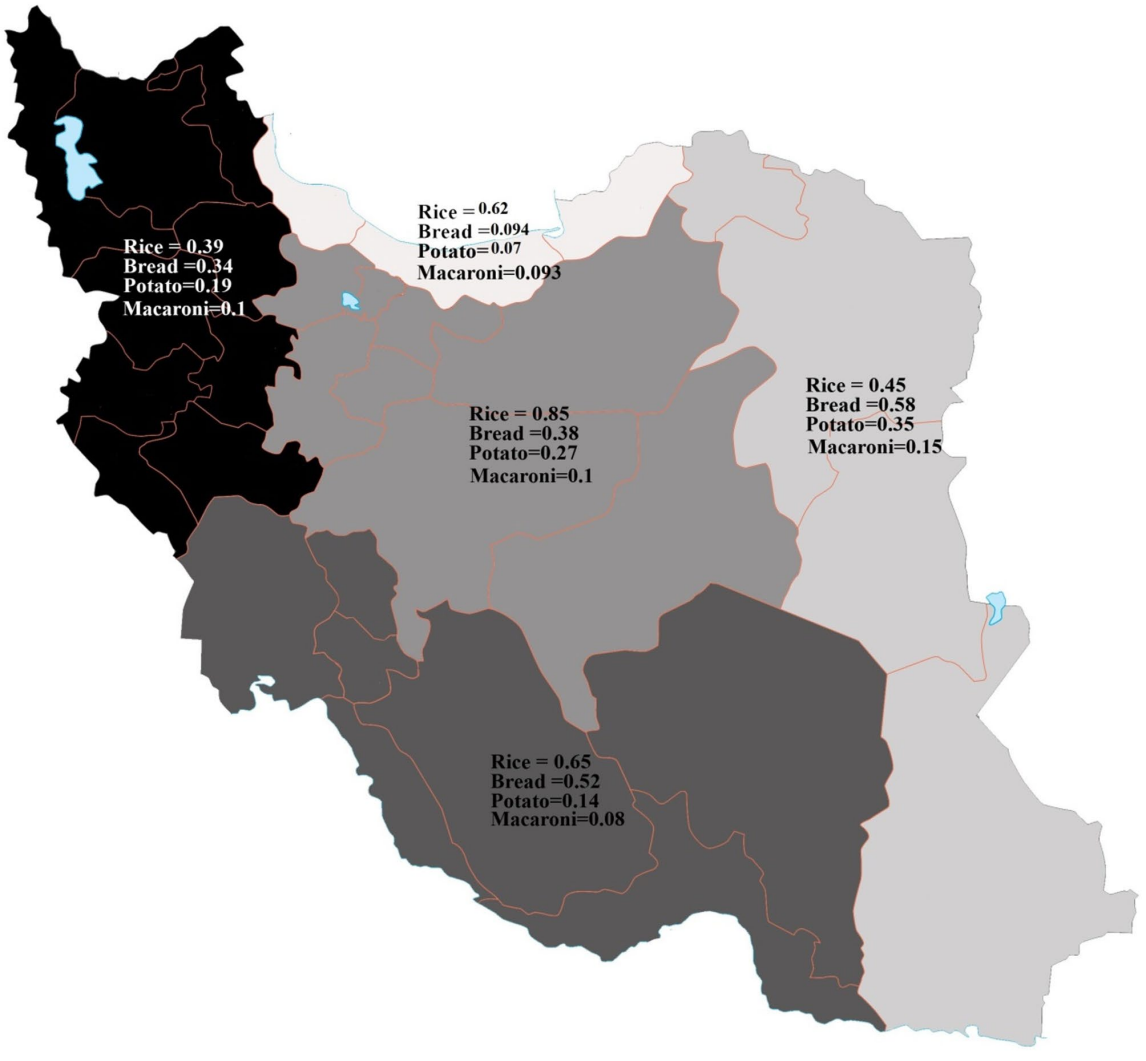
The per capita of Tah-digs in different regions of Iran Rice: *p* < 0.000; (Post-Hoc: East-West: 0.04, South –West: 0.001, West-Center: 0.000, East-Center: 0.000) Bread: *p* < 0.002 (Post-Hoc: East-West: 0.01) Potato: *p* < 0.000 (Post-Hoc: South- East: 0.02, East- West: 0.000, West- Center: 0.005) Macaroni: *p* < 0.021(Post-Hoc: South-West: 0.015)

Regional analysis (Fig. 1) indicated that bread and rice Tah-dig had the highest per capita consumption in the eastern and northern regions of Iran. In contrast, rice Tah-dig was most consumed in the central and southern regions. Statistically significant regional differences in Tah-dig consumption were identified (*p* < 0.05).

## Discussion

Per capita food consumption serves as a critical indicator of a nation’s total food supply and dietary patterns [21]. Numerous studies have underscored the central role of rice in the Iranian diet [22]. Globally, rice is a staple food for more than half of the world’s population, particularly in Asia, where it is considered indispensable [23]. Asia dominates global rice production—accounting for approximately 90% of total output—and also leads in consumption, with 84% of the world’s rice consumed within the region in the same year. Africa and South America followed with 7% and 3%, respectively. In 2013, the average per capita rice consumption in Asia was recorded at 77.8 kg [24] In the context of Iran, rice ranks as the second most consumed staple food after wheat. Notably, rice consumption in Iran is approximately seven times higher than in European countries [25, 26]. This elevated consumption pattern contributes to the predominance of rice Tah-dig as the most frequently prepared and consumed variant of Tah-dig nationwide.

Bread Tah-dig represents the second most commonly consumed variant of Tah-dig in Iran. As a staple food item, bread plays a pivotal role in shaping household dietary patterns [27]. Bread Tah-dig is typically prepared in conjunction with boiled rice, a method favored across many Iranian households. Findings from the present study reveal that bread Tah-dig is consumed at nearly twice the rate of potato Tah-dig, underscoring its cultural significance and dietary prevalence, particularly when paired with boiled rice.

National agricultural statistics identify potatoes as the third most significant crop in the country, following wheat and rice. The average annual per capita consumption of potatoes in Iran is approximately 45 kg [28]. In alignment with these broader consumption trends, findings from the present study indicate that potato Tah-dig ranks as the third most commonly consumed variant of Tah-dig. However, recent fluctuations in market prices and increased export activity have contributed to a noticeable decline in domestic potato consumption [28].

Macaroni-based Tah-dig exhibited the lowest per capita consumption among the variants examined in this study, with an average intake of 0.12 serving/day. This limited consumption may be attributed to declining consumer purchasing power, driven by the rising cost of processed food products in the Iranian market [29]. Moreover, the majority of study participants reported average to low household income levels, which likely contributed to the reduced prevalence of macaroni Tah-dig in their diets. It is also noteworthy that, in Iranian culinary practice, bread is commonly used as the Tah-dig base in the preparation of macaroni dishes.

Per capita consumption of bread and potato Tah-digs varied significantly across age groups. Elderly individuals reported lower intake of these variants, which may be attributed to age-related dental issues that limit the consumption of harder or crispier foods. Additionally, cooking duration was found to influence consumption patterns. Specifically, bread Tah-dig prepared with longer cooking times (30 and 45 min) was consumed more frequently than that prepared with a 15-minute duration, likely due to the enhanced crispiness and sensory appeal associated with extended cooking.

Oil usage also played a notable role in consumption behavior. When Tah-digs were prepared without oils such as olive, sesame, and canola, the highest per capita consumption was observed for bread, potato, and pasta Tah-digs, respectively. These contrasting findings may reflect regional and demographic variations among the study population. Moreover, recent increases in edible oil prices—driven by market liberalization—have had implications for both producers and consumers. This shift is anticipated to influence several dimensions, including consumer and producer welfare, per capita consumption patterns, and the overall supply of edible oils [30].

Previous research has demonstrated that the type of oil used in the preparation of Tah-dig significantly influences its chemical composition, particularly in relation to the formation of potentially harmful compounds such as acrylamide and PAHs [12, 15]. Adergani et al. (2020) investigated acrylamide concentrations in bread- and potato-based Tah-digs prepared with various oils. Their findings revealed that the highest acrylamide level (194.09 mg/kg) occurred in potato Tah-dig cooked with sunflower oil, whereas the lowest concentration (48.54 mg/kg) was detected in bread-based Tah-dig prepared with animal fat [15]. Similarly, PAH concentrations were found to be highest in Tah-digs prepared with canola and sesame oils, with bread-based variants exhibiting significantly greater PAH levels than potato-based counterparts [12]. The thermal cooking process plays a critical role in the formation of these contaminants. For instance, Shariatifar et al. (2024) reported that processed potato products such as chips contained higher median PAHs levels (19.28 µg/kg) compared to raw potatoes (6.31 µg/kg) [31]. Comparable results were observed in a study conducted by Karimi et al. (2022) in Kermanshah Province, Iran. The majority of participants (over 77%) reported consuming potato Tah-dig approximately five times per week [11]. Among the various types of Tah-dig analyzed, potato Tah-dig exhibited the highest acrylamide concentration, reaching 1.09 mg/kg. The mean acrylamide levels were determined to be 0.024 mg/kg in rice Tah-dig, 0.039 mg/kg in bread Tah-dig, and 0.714 mg/kg in potato Tah-dig. Notably, the maximum acrylamide content was recorded in potato Tah-dig (2.1 mg/kg), whereas the minimum was found in rice Tah-dig (0.024 mg/kg) [10].

Given the widespread consumption of Tah-dig in Iranian diets, these findings suggest a potential for elevated exposure to acrylamide and PAHs among the population [10, 12, 15]. The health risks associated with cooking practices that result in the formation of such toxins underscore the need for further investigation into safer preparation methods and public health interventions. Findings from the current study indicate that longer cooking durations for Tah-dig are associated with higher per capita consumption, which in turn correlates with increased formation of harmful compounds such as PAHs and acrylamide. These results underscore the need for public health education focused on the risks associated with prolonged cooking times and excessive oil use in Tah-dig preparation. Promoting shorter cooking durations and reduced oil quantities may help mitigate the formation of these toxic substances and contribute to safer dietary practices.

Regional variations in Tah-dig consumption across Iran reflect distinct cultural, agricultural, and culinary practices. Bread and rice Tah-dig exhibit the highest per capita consumption in the eastern and northern regions, respectively, while rice Tah-dig dominates in the central and southern areas. These consumption patterns are significantly shaped by regional customs and cultural preferences [32]. Northern Iran, as the primary hub for rice cultivation, demonstrates elevated per capita consumption of rice Tah-dig, consistent with its agricultural output. In contrast, wheat cultivation is more prevalent in the eastern provinces, contributing to the higher frequency of bread Tah-dig consumption in those areas. Taste preferences, climatic conditions, and economic factors further influence regional dietary choices.

Culinary techniques also vary geographically. In eastern Iran, rice is typically prepared by boiling and draining, a method that complements the use of bread as Tah-dig. Conversely, northern regions favor drained rice preparations, which align more closely with rice-based Tah-dig. These differences in preparation methods contribute to the observed regional disparities in Tah-dig consumption.

### Limitations

Primary limitation of this study was the incomplete responses to the questionnaire. One of the main limitations of the study was the limited responses in some cities despite the distribution of the questionnaire across multiple social platforms and sent to individuals in various regions of the country. Other limitations include sampling bias from self-reported data errors, uneven participation across regions, and the cross-sectional design’s inability to show causation and social media recruitment. Using social networks for recruiting participants has many advantages including access to broad audience, rapid recruitment, and reduced costs. But this method of recruitment offers some limitations. Firstly; social media users may not be representative of the wider population. For example, individuals who use social media actively usually are younger in age which can limit the generalizability of study results. A second limitation of using social network as recruitment platform is that not all population have access to the internet and such platforms. This issue would introduce a sampling bias into our study. It seems there is a need to combine social media recruitment with other methods to increase the representativeness of study sample.

## Conclusions

Tah-dig, a highly esteemed and culturally significant component of Iranian cuisine, is commonly served at social gatherings, ceremonial events, and in dining establishments across the country. Based on the findings of the present study, several key observations can be summarized. Rice and bread-based Tah-digs demonstrated the highest per capita consumption levels, surpassing those of potato and macaroni variants. This trend reflects both cultural preferences and dietary habits. Furthermore, the frequent use of oils such as olive, sesame, and canola in the preparation of rice and bread Tah-digs may contribute to their elevated consumption rates, suggesting a link between culinary practices and consumer behavior. The findings of this study highlight the critical need for targeted public health education addressing the risks associated with prolonged cooking durations and excessive oil usage in the preparation of Tah-dig. Encouraging the adoption of shorter cooking times and reduced oil quantities may serve as effective strategies to minimize the production of these toxic substances, thereby promoting safer dietary practices and reducing potential health risks. Given the potential formation of toxic compounds during the preparation of Tah-dig, further research is warranted to evaluate its safety and identify mitigation strategies. Continued investigation into cooking methods, ingredient selection, and exposure levels is essential to inform public health recommendations and promote safer culinary practices.

## Supplementary Information

Supplementary Material 1.

## Data Availability

Data is available upon reasonable request from the corresponding author.
