# Supplementary material for: Determining the per capita consumption of Tah-dig in the Iranian food table
Source: BMC Public Health. 2025 Dec 8;25:4229. doi: 10.1186/s12889-025-25551-6 (PMC12683913; doi:10.1186/s12889-025-25551-6)
Supplement: Supplementary file 1 — Supplementary Material 1. [file 12889_2025_25551_MOESM1_ESM.docx]

**Demographic questions**

1. Age (years):

2. Sex: Man ☐ woman☐

3. Monthly income status: good ☐ medium ☐ poor☐

4- Are you the person prepared the Tah-dig in the home? Yes☐ no☐

**If you are the correspond person for preparing the Tah-dig, Please, answer the following questions:**

1. What type of oil do you use for preparing the Tah-dig? Frying oil ☐ Hydrogenated fat ☐

Olive oil ☐ Sesame oil ☐ Canola oil ☐ Butter ☐ Ghee oil ☐ Corn oil ☐ sunflower oil ☐ Margarin☐

2. What is the cooking time of the Tah-dig?

15 minutes ☐ 30 minutes ☐ 45 minutes ☐

**Frequency and type of consuming Tah-dig**

**Generally how many times do you use the following Tah-digs?**

**Unit of measurement (serving size): one serving size is equal to a square of 10 centimeter × 10 centimeter**

| Tah-dig type | Number of Consumed serving size^★^ | Frequency of consumption | | | | | | | |
| --- | --- | --- | --- | --- | --- | --- | --- | --- | --- |
|  |  | Never | One time in a month | 1-3 times in a month | One time in a week | 2-4 times in a week | 5-6 times in a week | One time in a day | More than one time in a day |
| Bread |  |  |  |  |  |  |  |  |  |
| Potato |  |  |  |  |  |  |  |  |  |
| Rice |  |  |  |  |  |  |  |  |  |
| Macaroni |  |  |  |  |  |  |  |  |  |
| Tah-chin (a blend of rice, saffron, and yogurt) |  |  |  |  |  |  |  |  |  |
| Eggplant |  |  |  |  |  |  |  |  |  |
| Carrot |  |  |  |  |  |  |  |  |  |
| Squash |  |  |  |  |  |  |  |  |  |
| Spinach |  |  |  |  |  |  |  |  |  |
| cabbage |  |  |  |  |  |  |  |  |  |
| ^★^Serving size: in any time of consumption, how many squares of 10 cm × 10cm do you use? | | | | | | | | | |
